# Oral delivery of transgenic plant-derived HIV-1 p24 antigen in low doses shows a superior priming effect in mice compared to higher doses

**DOI:** 10.1186/1742-4690-9-S2-P336

**Published:** 2012-09-13

**Authors:** I Lindh, A Bråve, D Hallengärd, R Haddad, Å Strid, S Andersson

**Affiliations:** 1 School of Science and Technology, Örebro University, Örebro, Sweden; 2 Swedish Institute for Communicable Disease Control, Stockholm, Sweden; 3 Örebro University Hospital, Sweden, Örebro, Sweden

## Background

The gut associated lymphoid tissue (GALT) includes around two thirds of the total lymphoid system. CD4+ T-cells in the GALT are a main target for HIV during primary infection. Thus, immunization targetting GALT is likely to be of importance for an effective vaccine strategy. Transgenic plants expressing HIV antigens can reach GALT conveniently. This system allows multiple boosts, has simple logistics (no cold chain, no injections) and large production capacity.

## Methods

Three groups of mice were given extract from plant lines expressing HIV-1 p24 at (A) low level (20 ng/feeding); (B) high level (460 ng/feeding); (C) control (wild type, 0 ng). No adjuvant was included. The extracts were administered by gastric tube day 0, 14 and 28. On day 55 all mice were given an intramuscular (i.m.) boost with 10 micrograms of purified p24 antigen. Immune responses were determined by measurement of p24-antibodies in serum by ELISA.

## Results

The mice immunized by the low dose plant line (A) showed a higher systemic immune response after i.m. boost compared to the high dose group (B). The w.t. controls (C) had undetectable p24-responses. The responses in group A were 3 to 10 times higher (ELISA OD values) than in group B. Pre-boost antibody responses were at background levels in all groups. Preliminary analyses indicate a predomninant Th1-type response (antigen-specific IgG2a higher than IgG1).

## Conclusion

Simple and inexpensive means of vaccination are important in order to reach large numbers of people with effective vaccine regimens. The HIV-1 p24 low dose transgenic plant extracts given orally showed a superior priming effect in mice compared to the p24 high dose extracts. This could be an immunization method and route worth exploring further.

